# Effect of Descent Velocity upon Muscle Activation and Performance in Two-Legged Free Weight Back Squats

**DOI:** 10.3390/sports7010015

**Published:** 2019-01-07

**Authors:** Roland van den Tillaar

**Affiliations:** Department of Sport Sciences and Physical Education, Nord University, 7600 Levanger, Norway; roland.v.tillaar@nord.no; Tel.: +47-9766-2913; Fax: +47-7411-2001

**Keywords:** sticking point, sticking region, EMG

## Abstract

Background: The aim of this study was to investigate the effect of descent velocity during two-legged full back squats upon muscle activation and squat ascent performance. Methods: Eleven healthy resistance-training males (age: 24 ± 6 years, body mass: 89.5 ± 21.5 kg, height: 1.84 ± 0.10 m) performed 4-repetition maximum (4-RM) two-legged full squats with slow, normal, and fast descent phases. Kinematics and muscle activity of ten muscles divided into five regions were measured. Results: The main findings were that maximal and minimal velocity were lower and maximal velocity occurred later in the slow condition, while there was no difference in second peak velocity or ascent displacement when compared with the normal and fast conditions. Furthermore, no differences in muscle activation were found as an effect of the descent velocity. Conclusion: It was concluded that the slow descent velocity had a negative effect upon the ascent phase, because of the lower peak velocity and peak force increasing the chance of failure. The lower velocities were not caused by lower pre-activation of the muscles but were probably a result of potentiation and/or utilization of stored elastic energy and/or the stretch reflex.

## 1. Introduction

Training two-legged free weight back squats are widely used in many populations, with benefits including the improvement of maximal strength [1], power [2], and hypertrophy [3] of the lower limb. Many studies have investigated the effects of variations of squat depth [4], foot placement [5,6,7], instability [8,9], training status [10], and training intensity [11] during two-legged squats. These training variables vary depending on which specific goal the performers are aiming for [12]. For example, it has been recommended that lifting velocity in squats for athletes wishing to develop power is performed as fast as possible to elicit maximal power output and to train under an event-specific movement velocity [13,14]. However, movement velocity should be smooth and slow for conventional weight training designed for strength gaining or hypertrophy [15,16]. This smooth and slow movement was suggested to ensure constant muscle activation through the full range of motion and to maintain the correct lifting technique.

However, none of these studies have investigated the acute effect of the descent velocity upon the lifting performance. For the bench press, Sakamoto; Sinclair [17], Sakamoto; Sinclair [18] reported that with faster movement velocities more repetitions can be performed at given intensities. The authors suggested that enhanced utilization of the stretch–shortening cycle (SSC) was responsible for the increased numbers. In these studies, utilization of pre-activation [19,20], potentiation [21,22], stored energy, and/or stretch reflex were thought to explain the improved performance [17,18]. However, in these studies on movement velocity the descent and ascent movement velocities were prescribed to take equal amounts of time [17,18], but in regular lifting like in squats this is not the case and lifting time descent and ascent differs [23]. Furthermore, in squats with high intensity (>85% of 1-repetition maximum (1-RM)) a sticking region occurs [24,25], which is labelled as the weakest region of the lift in which the attempt often fails [26,27]. A faster descent velocity could perhaps elicit more of the described mechanisms during the stretch–shortening cycle in squats. This could result in higher ascent velocities at the start of the ascent, especially during the first 0.2–0.3 s of the lift [21]. Therefore, this faster velocity could decrease the first part of the sticking region and increase the chance of getting out of the sticking region during the lift [27]. However, no studies have investigated the effect of descent velocity upon the lifting performance in squats. In addition, no studies have included muscle activity measurements to gain more information behind the possible mechanisms involved in two-legged back squats that could explain their results.

Therefore, the aim of this study was to investigate the effect of descent velocity during two-legged full back squats upon muscle activation and squat ascent. It was hypothesized that higher descent velocity would have a positive effect upon the kinematics in the ascent phase; i.e., the sticking region would occur later and would be shorter, thereby the possibility to surpass the weakest link easier. Mechanisms like utilization of pre-activation, potentiation, stored energy, and stretch reflex are expected to be responsible for this effect. By investigating the electromyographic (EMG) development during the lifts it is possible to investigate if higher muscle (pre)activation is one of the mechanisms that is initiated by higher descent velocity or that other mechanisms cause an eventual positive effect in lifting performance.

## 2. Materials and Methods

We used a repeated-measures design to investigate the effect of descent velocity during back squats upon muscle activation and squat ascent. Each subject performed four repetitions at the assumed 4-RM weight at one of the prescribed descent velocities in a random order.

### 2.1. Subjects

Eleven healthy resistance-trained males (age: 24 ± 6 years, body mass: 89.5 ± 21.5 kg, height: 1.84 ± 0.10 m) with at least 2 years of free weight squat training experience participated in the study. The participants had a 4-repetition maximum (4-RM) weight of 129 ± 23 kg in back squats. Inclusion criteria were no injuries or pain in the last months before the test that could reduce their maximal performance and being able to lift 1.5 times their own bodyweight in back squats with good full squatting technique. Half of the participants were competing in powerlifting competitions at the national level. No resistance training of the legs was allowed in the 72 h preceding testing. All participants were informed verbally and in writing of the possible risks of the test and procedures. Before testing, written informed consent was given by the participants. The study was conducted with the approval of the Norwegian Centre for Research Data and conformed to the latest revision of the Declaration of Helsinki.

### 2.2. Procedures

To investigate the effects of descent velocity upon muscle activation and performance in the back squats, 4-RM free weight was used. The 4-RM weight was used as this is a typical training load to gain maximal strength [28]. Furthermore, it is also safer to conduct 4-RM than maximal 1-RM lifts in full squats due to the lower weights, and maximal 1-RM weights are not often used in regular resistance training [25].

The 4-RM and 1-RM weight was assumed by each participant based upon information given by each participant on 1-RM and 4-RM lifts performed in the previous six months. The two weeks before testing each participant was asked to train squats with different descent movement velocities to get familiar with these conditions. On the testing day, after a standardized warm-up, the assumed 4-RM was performed. The same warm-up protocol as Gomo; Van Den Tillaar [29] was used. The protocol was first a general warm-up, including as many repetitions as the participant wanted with just the barbell, then eight repetitions at 40% of the self-assumed one repetition maximum (1-RM_ass_), six reps at 60% of 1-RM_ass_, three reps at 70% of 1-RM_ass_, and two reps at 80% of 1-RM_ass_. Thereafter, the test started with four repetitions at the assumed 4-RM weight at one of the prescribed descent velocities.

The different velocities were based upon what the participant normally uses as descent velocity during the squats at that weight. Normal or preferred velocity was the velocity the participant usually uses during training with these weights. The slow velocity condition was instructed as just move slower than normal descent, while in the fast velocity condition it was instructed to move as fast as possible in descent while keeping control. It was not difficult for them to adjust to these three intended velocities since they were very experienced. The different descent velocities were given a random order and counter balanced to the participants to avoid fatigue, learning, or any other time-related effect affecting the results in a systematic way. The random order was determined by a random number generator.

Between each condition, participants were given five minutes rest to provide for optimal performance [30]. Furthermore, between each repetition, a pause of around 2–3 s was given in order that the participant could concentrate thus that each repetition was performed with maximal effort. No specific descent velocity was prescribed and each participant placed his feet in his own preferred position (to avoid extra stress upon the subject and increasing the ecological validity towards training) on a force plate (Ergotest Technology AS, Langesund, Norway) in which the position of the feet was measured. This position was then controlled and was identical in every later attempt. Then the lower position: Defined as the position in which the top surface of the legs at the hip joint is lower than the top of the knee, was determined using a protractor. A horizontal rubber band was used to identify this lower position during the tests, which the participants had to touch with their proximal part of the hamstring before starting the ascent [8,23,24,25]. The 4-RM back squats were performed with an Olympic barbell (2.8 cm diameter, length 1.92 m) with one spotter on each side of the barbell for safety. The participants bended from full knee extension in one of the prescribed tempos (slow, normal or fast), but controlled tempo until the back of their thigh touched the rubber band. Then, in the ascent part of the lift, the participants always had to move as fast as possible.

A linear encoder (ET-Enc-02, Ergotest Technology AS, Langesund, Norway) connected to the barbell measured the lifting time of the barbell with a resolution of 0.075 mm and counted the pulses at 10 ms intervals. The linear encoder and force plate were synchronized with wireless EMG recordings using a Musclelab^TM^ 6000 system and analyzed using Musclelab^TM^ v10.73 software (Ergotest Technology AS, Langesund, Norway). EMG activity of the vastus lateralis, vastus medialis, rectus femoris, gluteus maximus, gastrocnemius, soleus, semimembranosis, biceps femoris, external oblique, and erector spinae was measured with a sampling rate of 1000 Hz. Before placing the gel-coated self-adhesive electrodes (Dri-Stick Silver circular sEMG Electrodes AE-131, NeuroDyne Medical, Cambridge, MA, USA), the skin was shaved, abraded, and washed with alcohol. The electrodes (11 mm contact diameter and 2 cm center-to-center distance) were placed along the presumed direction of the underlying muscle fiber according to the recommendations by SENIAM [31]. The electrodes were placed on the right leg [8,25]. To minimize noise from the surroundings, the raw EMG signal was amplified and filtered using a preamplifier located close to the sampling point. The preamplifier had a common mode rejection ratio of 100 dB, the high-cut frequency at 500 Hz and low-cut frequency at 8 Hz. In order to compare EMG activity during the back-squat movement, EMG signals were bandpass filtered (20–500 Hz), rectified, and integrated (integrating moving average filter with 100 ms width) and converted to root mean square (RMS) for each region.

**Figure 1:**
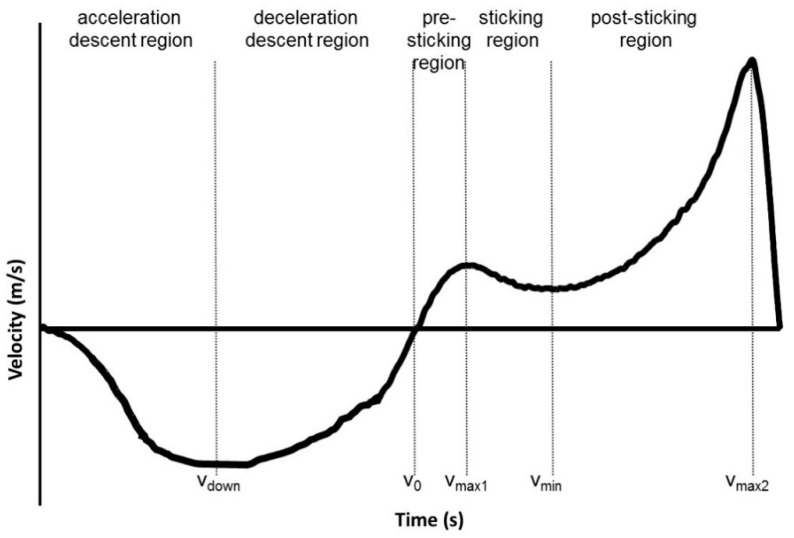
Typical vertical barbell velocity at maximal or near maximal attempts in squats with an acceleration descent, deceleration descent, pre-sticking, sticking, and post-sticking region, and the following events: Highest descent barbell velocity (v_down_), lowest position barbell (v_0_), first maximal barbell velocity (v_max1_), first located lowest barbell velocity (v_min_), and second maximal barbell peak velocity (v_max2_).

The different regions were based upon the different events and positions that occur during the lifts. The first region (Figure 1) was from the full extension position to the highest descent velocity (acceleration descent region). The second region was from the highest descent velocity (v_down_) to the lowest barbell point (deceleration descent region). The third region (pre-sticking region) was from the lowest barbell point (v_0_) until the first maximal barbell velocity (v_max1_); the next region (sticking region) was from the first maximal barbell velocity until the first located lowest vertical barbell velocity, also called the sticking point (v_min_); the last region, the post-sticking region, started at the first located lowest barbell velocity to the second maximal barbell peak velocity (v_max2_), which was also called the strength region [24,25,32]. Vertical displacement was measured in relation to the previous event like from v_down_ to v_0_ or from v_0_ to v_max1_. The velocity of the barbell was calculated by using a five-point differential filter with Musclelab^TM^ v10.73 software (Ergotest Technology AS, Langesund, Norway).

The RMS EMG of each region for each subject was calculated together with the velocities, displacement, and timing of the barbell at the different events and used for further analysis. Furthermore, the maximal vertical force was measured during each repetition in each condition and used for further analysis. Only repetitions two, three, and four were used for further analysis since earlier studies showed that in repetition one subjects perform with a significantly lower velocity than in the subsequent repetitions, which is caused by elaboration of the lifted weight [23].

### 2.3. Statistical Analysis

To assess differences between the three descent velocities in kinematics, a repeated 3 (descent velocity: Fast, normal, slow) × 3 (repetition: Two, three, and four) analysis of variance (two-way ANOVA) was used for each event/phase. To compare neuromuscular activity, a 3 (descent velocity: fast, normal, slow) × 3 (repetition: Two, three and four) × 5 (phase) analysis of variance (three-way ANOVA) was used for each of the 10 muscles. The post-hoc comparison was performed with Holm-Bonferroni. In cases where the sphericity assumption was violated, Greenhouse-Geisser adjustments of the *p*-values were reported. All results are presented as means ± standard deviations. Statistical significance was accepted at *p* ≤ 0.05. Effect size was evaluated with η^2^ (Eta partial squared), where a small effect was 0.01 < η^2^ < 0.06, a medium effect when 0.06 < η^2^ < 0.14 and a large effect when η^2^ > 0.14 [33]. Statistical analyses were performed in SPSS version 22.0 (SPSS Inc., Chicago, IL, USA).

## 3. Results

**Figure 2:**
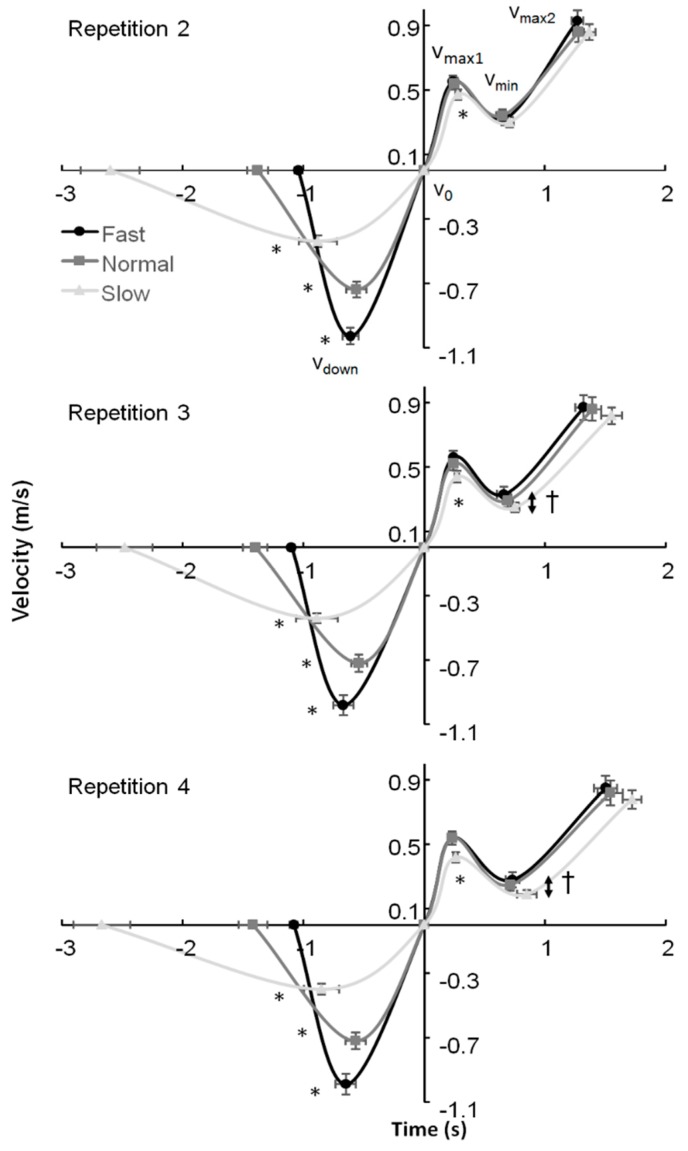
Mean (±SEM) velocity at highest descent barbell velocity (v_down_), lowest position barbell (v_0_), first maximal barbell velocity (v_max1_), first located lowest barbell velocity (v_min_) and second maximal barbell peak velocity (v_max2_) with their timing during repetitions two, three, and four of 4-RM squatting. * indicates significant difference with all other conditions in velocity and timing of this event at *p* ≤ 0.05. † indicates a significant difference between the slow and the fast condition in minimal velocity at *p* ≤ 0.05.

The lifted 4-RM load was 130 ± 23 kg and a clear sticking region in each of the repetitions was observed in each participant for all three descent velocities. The descent velocity was on average 0.3 m/s faster and slower in the fast and slow velocities, respectively than the preferred tempo (Figure 2). This resulted in significant differences in first maximal velocity and minimal barbell velocity (F ≥ 8.1, *p* ≤ 0.003, η^2^ ≥ 0.48) during the ascent phase. Furthermore, significant differences in the displacement of the barbell to v_down_ and from v_down_ to the deepest barbell point, peak force and timing of v_down_ and v_min_ during the lifts were identified (F ≥ 6.7, *p* ≤ 0.006, η^2^ ≥ 0.40). The post-hoc comparison showed that the peak v_down_ and the timing of this event were significantly different in each condition (from fast to normal to slow) in each repetition, while the v_max1_ event occurred significantly later in the slow condition compared to the other two conditions (Figure 2). The height of the barbell at which v_down_ occurred was at a greater distance from the ground than in the normal and slow conditions (Table 1), but the total displacement descent was the same among the conditions as shown by the higher displacement from v_down_ to v_0_ in the fast condition compared to the other two conditions (Table 1). This resulted in the same total descent displacement among the three conditions. In addition, post-hoc comparisons showed significantly lower peak forces, lower v_max1_ and v_min_ in the slow condition, and it took longer to reach v_max1_ in this condition in repetitions two and three compared to the other two conditions (Figure 2 and Table 1).

An effect of repetition was found for the velocity for v_min_ and v_max2_ and the timing of these two events (F ≥ 7.9, *p* ≤ 0.003, η^2^ ≥ 0.44). The post-hoc comparison revealed that the peak velocity at v_max2_ was significantly higher in repetition two compared to the other two repetitions in the fast and slow conditions. Peak v_min_ was also significantly higher in repetition two compared to the other two repetitions in the normal and slow conditions, while in the slow and fast conditions the peak v_min_ was significantly lower in repetition four compared to the other two repetitions (Table 1). In addition, it took a longer time to reach v_min_ and v_max2_ in repetition four compared with repetition two in all three conditions.

**Figure 3:**
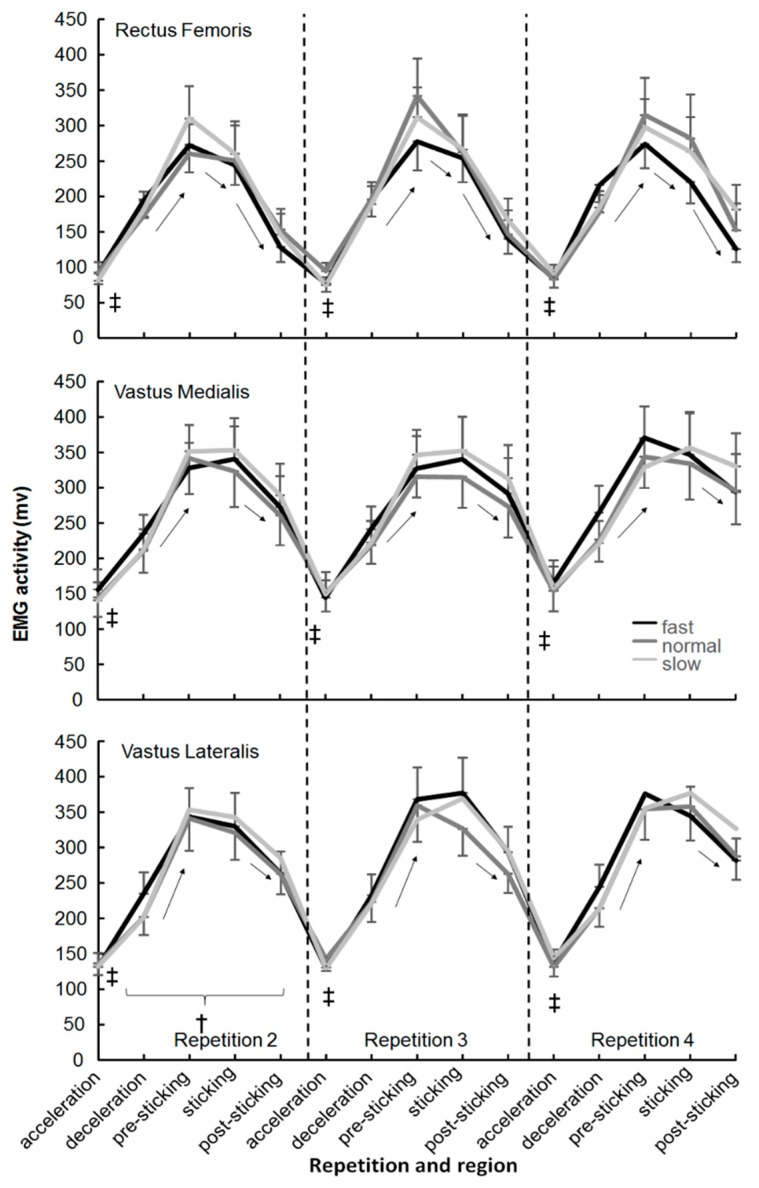
Mean (±SEM) root mean square (RMS) EMG activity of acceleration, descent deceleration, pre-sticking, sticking and post-sticking region of the quadriceps muscles during repetitions two, three and four of 4-RM squatting. ‡ indicates significant difference with all other regions at *p* ≤ 0.05. † indicates a significant difference with the other two repetitions at *p* ≤ 0.05. ↑ or ↓ indicates a significant difference in EMG activity between these two regions at *p* ≤ 0.05.

**Figure 4:**
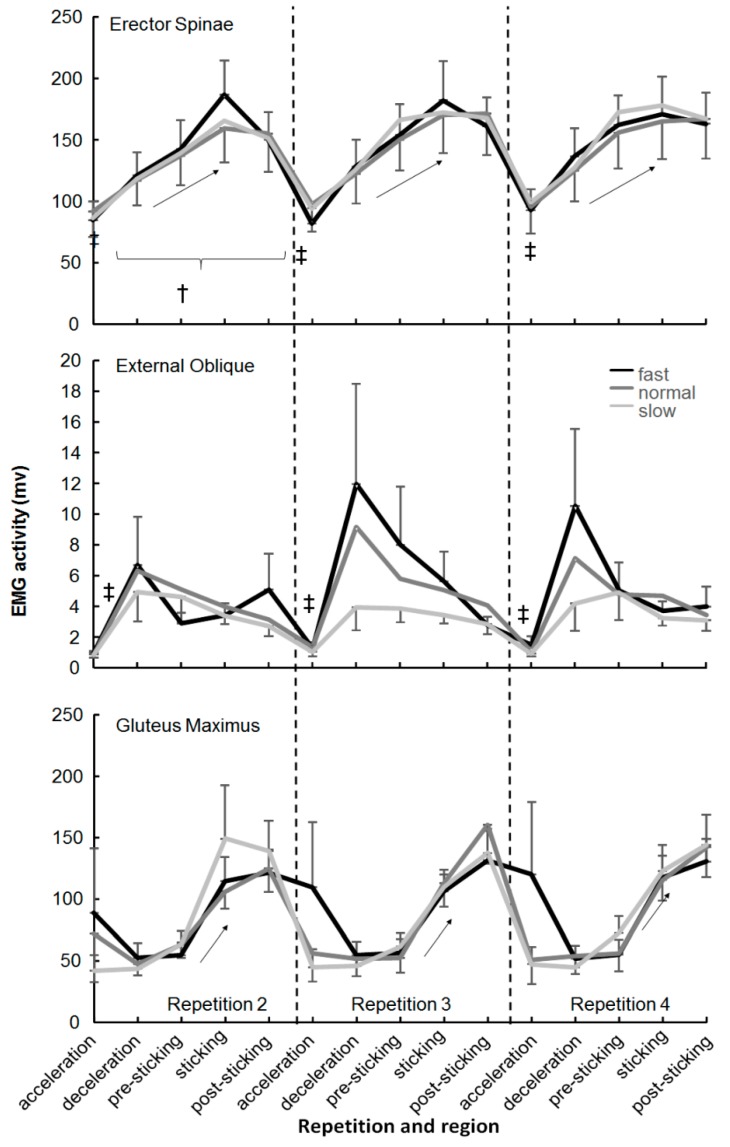
Mean (±SEM) root mean square (RMS) EMG activity of acceleration, descent deceleration, pre-sticking, sticking, and post-sticking region of the Erector Spinae, External Oblique, and Gluteus Maximus during repetitions two, three, and four of 4-RM squatting. ‡ indicates significant difference with all other regions at *p* ≤ 0.05. † indicates a significant difference with the other two repetitions at *p* ≤ 0.05. ↑ or ↓ indicates a significant difference in EMG activity between these two regions at *p* ≤ 0.05.

No significant effect of the descent moving barbell velocity was found in any of the muscles (F ≤ 2.1, *p* ≥ 0.152, η^2^ ≤ 0.24). Only a significant effect of repetition was found (F ≥ 6.6, *p* ≤ 0.007, η^2^ ≥ 0.42) for the lateral vastus and erector spinae (Figure 3 and Figure 4). The post-hoc comparison showed that EMG activity in repetition two was significantly lower than the other two repetitions in the deceleration, sticking, and post-sticking regions for the lateral vastus and for the erector spinae in almost every region.

**Figure 5:**
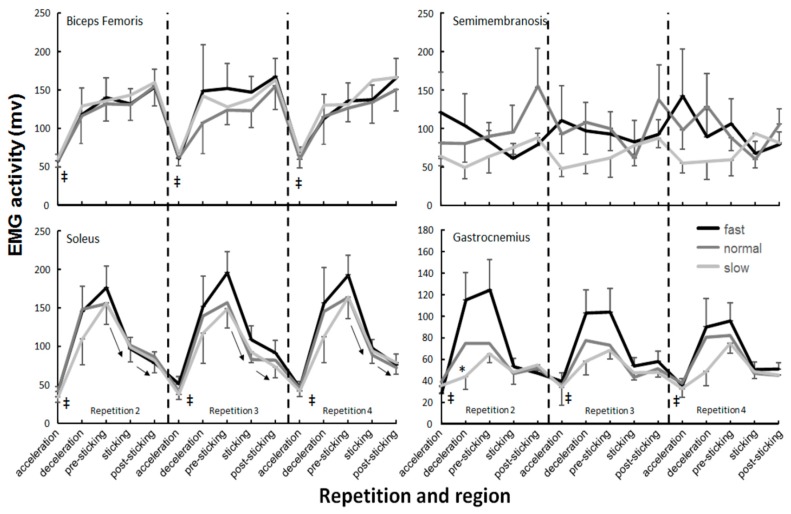
Mean (±SEM) root mean square (RMS) EMG activity of acceleration, descent deceleration, pre-sticking, sticking, and post-sticking region of the hamstrings and lower leg muscles during repetitions two, three, and four of 4-RM squatting. ‡ indicates significant difference with all other regions at *p* ≤ 0.05. ↑ or ↓ indicates a significant difference in EMG activity between these two regions at *p* ≤ 0.05. * indicates significant difference with the other two conditions in this region at *p* ≤ 0.05.

A significant effect of regions was found in every muscle except the semimembranosis (F = 0.4, *p* = 0.817, η^2^ = 0.04). The post-hoc comparison showed that in general, a significantly lower activity occurred in the acceleration for the descent region compared with the other regions (Figure 3, Figure 4 and Figure 5). Furthermore, that the external oblique, biceps femoris, and gastrocnemius muscles had similar EMG activity in the next four regions (Figure 4 and Figure 5). The three quadriceps muscles increased their activity until the pre-sticking region, where after it decreased again (Figure 3). Both gluteus maximus and erector spinae activity reached their highest EMG activity in the sticking region, where after it kept the same activity (Figure 4). The soleus muscle had the highest activity in the descent deceleration and pre-sticking region and decreased from there in each following region (Figure 5).

In addition, a velocity x region interaction effect was found for the gastrocnemius (F = 3.8, *p* = 0.001, η^2^ ≤ 0.36), i.e., the fast condition showed another development over the different regions than in the other two conditions (Figure 5). No other significant interaction effects were found.

## 4. Discussion

The purpose of this study was to investigate the effect of descent velocity during back squats upon muscle activation and squat ascent. The main findings were that v_max1_ and v_min_ were lower and the occurrence of v_max1_ was later in the slow condition, while there was no difference in v_max2_ or ascent displacement in the different events when compared with the other two conditions (Table 1). Furthermore, it seems that only the gastrocnemius muscle activation was affected by the descent velocity (Figure 5). The occurrences (barbell displacement and timing) of the different events (v_max1_, v_min_ and v_max2_), together with the velocity at v_min_ and v_max2_, in the last attempts in the present study were similar to van den Tillaar [24], who investigated the last attempt in 6-RM back squats, indicating that maximal attempts at these loads.

The descent moving velocity had an effect upon the sticking region events: Lower v_max1_ and v_min_ in the slow condition compared to the normal and fast ones. However, the sticking region did not occur later in the ascent displacement or become shorter, as hypothesized. The sticking region started at 0.09 ± 0.02 m and ended at 0.23–0.25 m in the ascent phase. The sticking region started later in the slow condition, but this was caused by the slower ascent movement in the pre-sticking region. The height at which the sticking region starts (0.09 m) was thereby reached later (Table 1). This indicates that the sticking region could be angle-specific thus that less force can be produced, also called a poor mechanical force production region [26,34,35]. In the present study, no joint angles or inverse kinetics were measured. However, the data are similar to that of van den Tillaar [24] who showed that the angles at which v_max1_ and v_min_ (sticking region) occurred were 65 ± 8, 71 ± 8 (ankle), 82 ± 18, 103 ± 15 (knee), and 84 ± 11, 101 ± 11 (hip) degrees. At these angles, probably due to the large external moments of the ankle, knee, and hip joints, less force can be produced. Van den Tillaar [24] showed that the start of the sticking region (v_max1_) coincided with the timing of the first peak angular velocity of the plantar flexion and knee extension, while the timing of v_min_ was at around the same time as the minimal plantar flexion and knee extension angular velocity (end of the sticking region). Furthermore, he found that the time of occurrence of the v_max2_ of the barbell matched with the peak angular velocity of the hip extension and the second peak of the plantar flexion and knee extension, which resulted in a higher second peak velocity (0.54 vs. 0.83 m/s). This was visible in the present study by the highest activity of the calf (Figure 5) and quadriceps muscles (Figure 3) during the pre-sticking and sticking regions, while the gluteus maximus had the highest activity in the post-sticking region (Figure 4).

No differences in muscle activation between the three conditions were found. Therefore, the differences in kinematics (peak velocities at v_max1_ and v_min_) in the fast and normal conditions were probably not caused by an increased pre-activation of muscles during the descent phase (Figure 3, Figure 4 and Figure 5) as Bobbert and colleagues [19,20] suggested as a mechanism that occurs in a stretch–shortening cycle. It is more likely that the higher velocities at v_max1_ and v_min_ in these two conditions were caused by potentiation [21,22], which is initiated by the higher peak force at the deepest barbell position in these two conditions (Table 1). Walche; Wilson; Ettema [21] showed that the first 0.3 s during the ascent in squats can be enhanced by potentiation and thus cause a higher v_max1_ as this happens in the first 0.3 s of the ascent phase. Due to this higher v_max1_, the v_min_ in these conditions was also higher, since it decreased by approximately the same amount (0.23 m/s) as in the slow condition (Table 1). Other mechanisms that could explain the higher v_max1_ and v_min_ in the fast and normal conditions are the utilization of stored elastic energy and/or the stretch reflex [17,18], which was indicated by the higher peak forces found in the normal and fast conditions.

The different peak descent velocities occurred differently in time in the descent acceleration region (doubled from condition to condition: 0.4, 0.8, 1.6 s) between each condition. However, this only had an effect on the barbell height in the fast condition, which was much higher in the descent movement compared to the normal and slow conditions (Table 1). Since the time in the deceleration region was approximately the same for all three conditions, and the peak velocities were different, this caused a higher peak force in the lowest barbell position in the fast and normal conditions compared with the slow condition. Since the highest peak descent velocity of the barbell in the fast condition was much higher in the lifting trajectory, it had the possibility to decelerate over a longer descent trajectory than in the normal condition, which enabled similar peak forces in these two conditions (Table 1). Surprisingly, the differences in peak forces at the lowest barbell point did not result in muscle activation differences in the deceleration region between the three conditions. An explanation for this could be that due to the slow descent movement in the slow condition, the muscles are under tension for longer, which could result in relatively higher activation of these muscles. This could equalize the possible lower muscle activation due to lower peak forces at the deepest barbell point in the squat.

There were not many effects of repetition found on the kinematics and muscle activity among the three conditions. Only v_min_ and v_max2_ decreased and it took longer to reach these two events (longer in sticking and post-sticking region) independently of the condition, which was in line with van den Tillaar; Andersen; Saeterbakken [23] who showed that fatigue causes increased total lifting time and decreased peak velocity. That the pre-sticking region was not affected by repetition indicated that mechanisms involved in this part of the lift are independent of fatigue. The increasing demand during repetitions was only visible by the increased muscle activation of the vastus lateralis and erector spinae, indicating that these two muscles become more important when fatigue occurs.

It seems that only gastrocnemius muscle activity was affected by the descent velocity conditions (Figure 5). It was found that in the fast condition, muscle activation decreased per repetition, while it increased in the normal and slow conditions. This was especially visible in the deceleration and pre-sticking regions. During the deceleration region, the gastrocnemius works eccentrically to avoid too much dorsal flexion. By increasing descent velocity, activation has to increase. Due to the high descent velocity, this muscle may become more fatigued than in the normal and slow conditions, which may lead to decreased activity in these regions in later repetitions. Furthermore, greater involvement of the ankle (altered kinematics) at the end of the downward phase could be caused by the higher descent velocity.

However, as a limitation of the present study, no 3D kinematics were conducted to investigate if the changed gastrocnemius activity was caused by different dorsal and plantar flexion movements. Furthermore, these 3D kinematical analyses, together with inverse kinetics, could give more information about the cause of this sticking region and the effect of descent velocity upon the joint movements in the lifting movement in free weight back squats. Future studies should include 3D measurements and perform inverse kinetics to establish more detailed information about these variables.

## 5. Conclusions

In summary, it can be concluded that slow descent velocity caused lower peak force and velocities at v_max1_ and v_min_ and it takes longer in the sticking and post-sticking regions. It thereby increases the chance of failure, since the sticking region is the weakest region of the lift [27]. The lower velocities were not caused by lower pre-activation of the muscles but were probably a result of potentiation, utilization of stored elastic energy, and/or the stretch reflex. Based on the present findings it is advised to athletes and coaches that the descent movement in back squats would be as high as possible, without losing control. This helps in the ascending part, to overcome the sticking region quicker and thereby decrease the chance of failure of the lift.

## Figures and Tables

**Table 1:** Mean (±S.D.) barbell velocity, displacement, and time interval at maximal descent barbell velocity (v_down_), lowest barbell point (v_0_), first maximal barbell velocity (v_max1_), minimal vertical barbell velocity (v_min_) and second peak barbell velocity (v_max2_) during the 2-legged squat movement.

Events	Interval (s)			Velocity (m/s)			Displacement Barbell (m)			Maximal Force (F)		
	Fast	Normal	Slow	Fast	Normal	Slow	Fast	Normal	Slow	Fast	Normal	Slow
*Repetition 2*												
V_down_	0.43 ± 0.11 *	0.82 ± 0.29 *	1.72 ± 0.80 *	−1.03 ± 0.17 *	−0.74 ± 0.15 *	−0.44 ± 0.12 *	0.26 ± 0.10 *	0.38 ± 0.16	0.40 ± 0.20			
V_0_	0.61 ± 0.22	0.56 ± 0.27	0.88 ± 0.52				0.41 ± 0.09 *	0.28 ± 0.15	0.25 ± 0.16	3195 ± 792	3067 ± 686	2778 ± 559 *
V_max1_	0.24 ± 0.07	0.25 ± 0.06	0.28 ± 0.07 *	0.55 ± 0.12	0.54 ± 0.10	0.47 ± 0.11 *	0.10 ± 0.04	0.09 ± 0.03	0.09 ± 0.03			
V_min_	0.43 ± 0.12	0.40 ± 0.16	0.43 ± 0.11	0.32 ± 0.13	0.34 ± 0.14	0.30 ± 0.11	0.16 ± 0.03	0.15 ± 0.04	0.15 ± 0.03			
V_max2_	0.60 ± 0.16	0.63 ± 0.15	0.66 ± 0.17	0.93 ± 0.21	0.86 ± 0.23	0.86 ± 0.17	0.31 ± 0.03	0.31 ± 0.03	0.31 ± 0.04			
*Repetition 3*												
V_down_	0.43 ± 0.09 *	0.86 ± 0.34 *	1.59 ± 0.77 *	−0.98 ± 0.21 *	−0.72 ± 0.18 *	−0.44 ± 0.10 *	0.24 ± 0.10 *	0.39 ± 0.15	0.39 ± 0.19			
V_0_	0.67 ± 0.27	0.54 ± 0.22	0.89 ± 0.58				0.42 ± 0.08 *	0.27 ± 0.14	0.26 ± 0.17	3254 ± 842	3070 ± 743	2772 ± 551 *
V_max1_	0.24 ± 0.06	0.24 ± 0.05	0.27 ± 0.07 *	0.56 ± 0.14	0.52 ± 0.14	0.44 ± 0.12 *	0.09 ± 0.03	0.09 ± 0.02	0.08 ± 0.02			
V_min_	0.42 ± 0.19	0.45 ± 0.14	0.48 ± 0.12	0.33 ± 0.16	0.29 ± 0.13 ↓	0.25 ± 0.10 ↓†	0.16 ± 0.04	0.16 ± 0.03	0.15 ± 0.03			
V_max2_	0.66 ± 0.24	0.70 ± 0.24	0.80 ± 0.28 ↓	0.87 ± 0.25 ↓	0.86 ± 0.24	0.82 ± 0.17 ↓	0.30 ± 0.03	0.31 ± 0.04	0.32 ± 0.05			
*Repetition 4*												
V_down_	0.43 ± 0.11 *	0.85 ± 0.40 *	1.82 ± 0.78 *	−0.99 ± 0.20 *	−0.72 ± 0.17 *	−0.40 ± 0.12 *	0.26 ± 0.12 *	0.38 ± 0.17	0.42 ± 0.17			
V_0_	0.65 ± 0.27	0.57 ± 0.28	0.85 ± 0.48				0.41 ± 0.10 *	0.29 ± 0.15	0.22 ± 0.14	3203 ± 880	3112 ± 741	2690 ± 474 *
V_max1_	0.23 ± 0.05	0.23 ± 0.05	0.26 ± 0.06 *	0.54 ± 0.14	0.54 ± 0.14	0.42 ± 0.11 *	0.09 ± 0.02	0.09 ± 0.02	0.08 ± 0.03			
V_min_	0.50 ± 0.20 ↓	0.48 ± 0.12 ↓	0.59 ± 0.27 ↓	0.28 ± 0.15 ↓↓	0.25 ± 0.13 ↓	0.19 ± 0.08 ↓↓†	0.17 ± 0.04	0.16 ± 0.02	0.15 ± 0.05			
V_max2_	0.77 ± 0.31 ↓	0.83 ± 0.35 ↓	0.87 ± 0.26 ↓	0.85 ± 0.26 ↓	0.82 ± 0.26	0.78 ± 0.19 ↓	0.31 ± 0.04	0.32 ± 0.04	0.31 ± 0.05			

V_down_ = highest descent barbell velocity, V_0_ = lowest barbell point, V_max1_ = first maximal barbell velocity, V_min_ = the first located lowest vertical barbell velocity, also called the sticking point, V_max2_ = second maximal barbell peak velocity. * Indicates significant difference from the other two conditions at this event at a *p* < 0.05 level. † Indicates significant difference from the fast condition at this event at a *p* < 0.05 level. ↓ Indicates a significant difference between this repetition and repetition 2 at his event at a *p* < 0.05 level. ↓↓ Indicates significant difference between this repetition and repetitions 2 and 3 at his event at a *p* < 0.05 level.
